# Antimicrobial Resistance in Commensal *Escherichia coli* from Pigs during Metaphylactic Trimethoprim and Sulfamethoxazole Treatment and in the Post-Exposure Period

**DOI:** 10.3390/ijerph120202150

**Published:** 2015-02-16

**Authors:** Justyna Mazurek, Ewa Bok, Michał Stosik, Katarzyna Baldy-Chudzik

**Affiliations:** Department of Molecular Biology, Faculty of Biological Sciences, University of Zielona Góra, Monte Cassino 21b, 65-561 Zielona Góra, Poland; E-Mails: e.bok@wnb.uz.zgora.pl (E.B.); m.stosik@wnb.uz.zgora.pl (M.S.); k.baldy-chudzik@wnb.uz.zgora.pl (K.B.-C.)

**Keywords:** *E. coli*, food production animals, metaphylaxis, antimicrobial resistance, resistance genes, transcription analysis, resistance genes expression, integrons, integron promoters

## Abstract

The prevalence of trimethoprim (TMP) and sulfamethoxazole (SMX) resistance in commensal *E. coli* from pigs was tested in this study. *E. coli* was derived from three groups of piglets in successive stages of metaphylactic therapy and from two groups of sows 10 and 18 weeks after the treatment. MIC values of TMP and SMX were determined for a total of 352 strains. The presence of resistance genes (*dfrA1*, *dfrA5*, *dfrA7*, *dfrA12*, *dfrA17*,* sul1*,* sul2*,* sul3*) and class 1 and 2 integron-associated *dfrA* gene cassettes was tested. Resistance to TMP was very high during the administration of the antimicrobial (from 97 to 100%) and amounted to 86% and 69% in the post-exposure period; MIC > 32 mg/L. The isolates from all groups of pigs were resistant to sulfamethoxazole, with MIC > 1028 mg/L. The *dfrA1* and* sul1* genes (as part of integrons) *dominated* in *E. coli* from piglets, but the *dfrA12* and *sul1* genes were prevalent in *E. coli* from sows. Coexistence of the different *dfrA* genes was detected in 71 isolates from all groups of swine. Transcription analysis revealed that most of these genes were not transcribed, particularly gene cassettes of class 1 integrons. The research revealed a high level of resistance associated with the metaphylactic treatment, persistence and circulation of resistance in bacterial populations. Diverse genetic background with multiple and not transcribed resistance genes was observed.

## 1. Introduction

The use of antimicrobials in the treatment of diseases of food-producing animals began in the mid-1940s. In the early 1950s, antimicrobials were introduced to commercial feed for cattle, pigs and poultry [1]. The worldwide use of antimicrobials in veterinary medicine is very high. For example, according to the recent Food and Drug Administration report, in 2012 in the United States, sales and distribution of antimicrobials for use in food-producing animals was over 14 thousand tons [2]. Although in European Union sales of veterinary antimicrobial agents is lower, it still occurs at a significant level [3]. The increase in demand for livestock products and the development of farming, nutrition and management have led to changes in the animal production systems. The intensification of food production systems rules out tolerance of disease outbreaks; thus, various antimicrobial drugs are administered for the prevention of diseases. In pig farming, treatment is often applied in young piglets. The mortality of piglets is high in the most critical periods of growth, during the first weeks of life and immediately after weaning piglets from a sow. The post-weaning stress and the related immunocompromised state promotes the development of bacterial infections such as respiratory infections, diarrhea and enteritis [4,5,6]. Antimicrobials are administered in the control treatment called metaphylaxis in order to avoid the spread of disease. Trimethoprim and sulfonamides in combination are antimicrobial agents that are commonly used in such cases. They are synthetic drugs that act as competitive inhibitors of the successive steps of the folic acid synthesis and are effective against a wide range of Gram-positive and Gram-negative microorganisms [7,8].

Due to the spatially limited environment and repetitive production scheme, large breeding farms form closed enclaves, where the pool of the resistant microorganisms and resistance genes can be accumulated and circulate between animals and their environment. It is interesting to what extent the supply of antimicrobials results in the development of resistance and whether after the cessation of antimicrobial pressure resistance decreases. This study concerns the relationship between TMP-SMX use in the metaphylactic treatment of post-weaning piglets and the resistance of commensal *E. coli* from pigs. The research include the determination of the phenotypic and genotypic TMP-SMX resistance prevalence, class 1 and 2 integrons and plasmids characterisation. It also concerns the study of resistance gene transcription and the correlation of the resistance genes transcription with integron and non-integron location of the genes.

## 2. Experimental Section

### 2.1. Research Material

The research material was derived from a pig breeding farm in the western part of Poland, the Lubuskie Voivodeship. The farm is one of the largest in the region, with an annual production that amounts to 60,000 pigs. The farm works in a closed system. It includes the entire production cycle from sows to piglets to porkers. Five groups of pigs, which were subjected to the same metaphylaxis program, were chosen for the research. The groups consisted of individuals of different age. Fecal samples were taken from 6-week-old piglets, after the first week of treatment (Piglets 1; P1), from a 7-week-old cohort taking antimicrobials for 2 weeks (Piglets 2; P2), and an 8-week-old cohort taking antimicrobials for 3 weeks (Piglets 3; P3). The sampling also covered two herds of sows that were subjected to the same metaphylaxis program in the past. They were 10 (Sows 1; S1) and 18 weeks (Sows 2; S2) after the end of treatment and did not take any other antimicrobials before the time of sampling. Sows 1 were 4.5-months-old gilts, Sows 2 were 6-months-old first parity sows. The medical program was applied after the diagnosis of colibacillosis. The antimicrobials were administered in the form of medicated fodder which contained trimethoprim/sulfamethoxazole, 15–30 mg of active substance per 1 kg of body weight per day.

### 2.2. E. coli Isolation and Selection of Non-Identical Strains

Fecal samples were plated on mFC chromogenic agar (Merck, Darmstadt, Germany), and after incubation at 44 °C, 2 to 3 randomly selected blue colonies were cultured on MacConkey agar (Merck). Next, typical lactose fermenting colonies were identified by biochemical IMViC testing for identification of *E. coli*. Selection of non-identical *E. coli* isolates was performed via BOX-PCR fingerprint analysis [9,10,11] and phylogenetic grouping (identification of the main phylogenetic groups: A, B1, B2 and D) [12]. *E. coli* isolates were considered as non-identical (individual strains) when they demonstrated less than 80% genomic similarity in BOX-PCR fingerprint analysis and belonged to different phylogenetic groups (results not shown). A total of 352 *E. coli* strains (1 strain per animal) were selected for further investigations: 59 from Piglets 1, 72 from Piglets 2, 82 from Piglets 3, 77 from Sows 1 and 62 from Sows 2.

### 2.3. Antimicrobial Susceptibility Testing

*E. coli* isolates were tested for their antimicrobial susceptibility to the antimicrobials which were administered to the pigs in the metaphylactic treatment. Minimum inhibitory concentrations (MIC, mg/L) of trimethoprim and sulfamethoxazole were determined by the broth microdilution method using Sensititre plates for veterinary application (TREK Diagnostic Systems, Oakwood Village, OH, USA), in the range 0.5–32 mg/L for TMP and 8–1024 mg/L for SMX. The results were interpreted according to the epidemiological MIC cut-off values set by EUCAST (EFSA) with the breakpoint for resistance to trimethoprim set at > 2 mg/L, and sulfamethoxazole > 64 mg/L [13,14]. *E. coli* ATCC 25922 was used as a susceptibility control strain.

### 2.4. Resistance Genes Detection 

Five TMP resistance genes: *dfrA1*, *dfrA5*, *dfrA7*, *dfrA12*, *dfrA17*, and three SMX resistance genes: *sul1*,* sul2* and *sul3*, were chosen for the genotypic studies. The presence of all *dfrA* genes was detected by multiplex PCR designed by Grape [15]. Detection of *sul* genes was carried out using PCR as reported previously [16]. Bacterial thermal lysates were used as a DNA template. Positive and negative controls were included in all PCR arrays.

### 2.5. Detection and Characteristics of Class 1 and Class 2 Integrons

The presence of class 1 and class 2 integrons was analyzed by PCR amplification of integrase genes (*int1*,* int2*), variable regions containing gene cassettes (c1gc, c2gc) and the 3ꞌ conserved region of the class 1 integron (*qacEΔ1*, *sul1*) [17]. Randomly chosen, same-size PCR products of the variable regions of integrons were purified (Isolate II PCR and Gel Kit; Bioline, London, UK) and sequenced (Genomed, Warsaw, Poland). Characterization of the class 1 integron gene cassette promoter, including the Pc-P2 region, was performed by amplification and sequencing with the designed primers: PromI1-F 5ꞌ-AGGACAGAAATGCCTCGACT-3ꞌ and PromI1-R 5ꞌ-CCCGAGGCATAGACTGTACA-3ꞌ, located in *intI1* and *attI* sequences, respectively.

### 2.6. Plasmid Characteristics

Plasmids were isolated using the Plasmid DNA purification Kit (Thermo Scientific, Waltham, MA, USA) according to the manufacturer’s instructions and separated electrophoretically. Plasmid size was estimated in relation to the size of the plasmids isolated from *E. coli* V517 reference strain, using the BioGene V.99.03 system (Vilber Lourmat, Collegien, France). PCR-based replicon typing was performed for the recognition of the plasmid incompatibility group replicon types: F, FIA, FIB, FIC, B/O, X, Y, N, P, W, T, A/C, HI1, HI2, I1-Ic, L/M, K and FII [18].

### 2.7. Real-Time PCR Assays with Reverse Transcription

The analysis of *dfrA* gene transcription in *E. coli* isolates with the co-existing *dfrA* genes was performed by the RT-PCR method. LB broth (Merck) was inoculated by overnight bacterial culture and grown at 37 °C to the logarithmic phase (OD_600_ = 0.8). Each strain was cultured in the presence of TMP (at a concentration under the individual MIC value: 32 mg/L) and in the absence of antimicrobial. The RNA was extracted using the SV Total RNA Isolation System (Promega, Madison, WI, USA) and quantitated based on absorption at 260 nm, and purity was determined by the ratio of the absorption values at 260 and 280 nm. Residual DNA was digested with DNase (Roche, Basel, Switzerland). The reverse transcription and real-time analysis were conducted with the DyNAmo SYBR Green 2-Step qRT-PCR Kit (Thermo Scientific) according to the manufacturer’s recommendations. Random hexamers were used to prepare cDNA from 0.5 µg of total RNA for each sample. Real-time PCR reactions were performed on a Rotor-Gene 3000 (Corbett Research, Sydney, Australia) in 20 µL volumes, containing 10 µL of 2× Master mix buffer, 0.5 µM of each primer and 1 µL of cDNA template. In each analysis, a no-template control (NTC) and a no-reverse transcriptase control (-RT) were included, and no transcription profiles were observed before the onset of 40 cycles of PCR. Positive controls of the transcription were performed by using primers specific for the gene encoding D-glyceraldehyde-3-phosphate dehydrogenase A (*gapA*). The positive RT-PCR signal was defined as a threshold cycle (Ct) value <35. All transcription assays were done in duplicate, using RNA from a single isolation.

## 3. Results and Discussion

### 3.1. Antimicrobial Susceptibility of Isolates

The majority of the isolates were resistant to the tested antimicrobials. The TMP resistance was 97.3% and 100% in the strains from Piglets 1, 2 and 3 during the metaphylaxis The prevalence of the resistant strains from pigs in the post-exposure period was lower: 86% and 69% for Sows 1 and Sows 2, respectively (Table 1). All the strains from piglets during the medical program were resistant to SMX. Also all the tested *E. coli* from Sows 1 and 92% of strains from Sows 2 were resistant to SMX (Table 1). The MIC values were > 32 mg/L for TMP and > 1024 mg/L for SMX for all of the tested *E. coli* from pigs during the metaphylaxis and for most of resistant *E. coli* from pigs in the post-exposure period.

**Table 1:** Resistance to trimethoprim and sulfamethoxazole of *E. coli* isolated from groups of pigs during metaphylaxis (P1–P3: antimicrobial pressure present) and in post-exposure period (S1, S2: antimicrobial pressure absent).

Group of Pigs	Antimicrobial Pressure	No. (%) of *E. coli* Resistant To	
		Trimethoprim	Sulfamethoxazole
P1 (*n* = 59)	Present	58 (97.3)	59 (100)
P2 (*n* = 72)	Present	71 (100)	71 (100)
P3 (*n* = 82)	Present	81 (100)	81 (100)
S1 (*n* = 77)	Absent	67 (86)	77 (100)
S2 (*n* = 62)	Absent	43 (69)	58 (100)
Total (*n* = 352)		321 (92)	352 (100)

### 3.2. Prevalence of Resistance Genes

All 352 strains were tested for the presence of TMP and SMX resistance determinants. The *dfrA1* gene was the most commonly detected trimethoprim resistance gene in *E. coli* from all groups of piglets. The *dfrA1* and *dfrA12* genes occurred with similar frequencies in strains from two groups of sows. The *dfrA7* and *dfrA5* genes were detected with a lower incidence in all groups (Table 2).

The *dfrA17* gene was not identified in any of the tested isolates. The sulfamethoxazole resistance gene *sul1* dominated in *E. coli* from piglets and the *sul3* gene dominated in *E. coli* from sows. The *sul2* gene occurred with lower frequencies in *E. coli* from all groups (Table 2). The coexistence of multiple resistance genes in single strains was also found (Table 3).

**Table 2:** Prevalence of trimethoprim and sulfamethoxazole resistance genes in *E. coli* isolated from groups of pigs during metaphylaxis and in post-exposure period.

Group of Pigs	Antimicrobial Pressure	No. (%) of *E. coli* with Detected						
		TMP Resistance Genes				SMX Resistance Genes		
		*dfrA1*	*dfrA5*	*dfrA7*	*dfrA12*	*sul1*	*sul2*	*sul3*
P1	Present	47	3	4	27	32	7	23
(*n* = 59)		(80)	(5)	(7)	(46)	(54)	(12)	(39)
P2	Present	63	8	8	9	56	8	23
(*n* = 72)		(88)	(11)	(11)	(13)	(78)	(11)	(32)
P3	Present	49	12	14	22	48	25	26
(*n* = 82)		(60)	(15)	(17)	(27)	(59)	(30)	(32)
S1	Absent	25	4	3	27	19	12	28
(*n* = 77)		(40)	(6)	(5)	(44)	(24)	(15)	(36)
S2	Absent	40	4	10	37	15	2	25
(*n* = 62)		(51)	(5)	(13)	(47)	(24)	(3)	(40)

**Table 3:** Number of *E. coli* from groups of pigs during metaphylaxis (A+) and in post-exposure period (A−), with combination of coexisting trimethoprim resistance genes.

Coexisting TMP Resistance Genes				P1	P2	P3	S1	S2
				(*n* = 59)	(*n* = 72)	(*n* = 82)	(*n* = 77)	(*n* = 62)
				A+	A+	A+	A−	A−
*dfrA1*	*dfrA12*			15	5	2	6	9
*dfrA1*	*dfrA5*			0	6	2	0	1
*dfrA1*	*dfrA7*			0	3	5	1	1
*dfrA5*	*dfrA7*			1	0	2	0	0
*dfrA5*	*dfrA12*			0	0	1	0	2
*dfrA7*	*dfrA12*			0	0	0	1	0
*dfrA1*	*dfrA5*	*dfrA7*		0	1	1	0	0
*dfrA1*	*dfrA7*	*dfrA12*		1	0	0	0	4
*dfrA1*	*dfrA5*	*dfrA7*	*dfrA12*	1	0	0	0	0
Total				18	15	13	8	17

### 3.3. Integron Characteristics

Class 1 integrons containing *int1-c1gc-qacΔE-sul1* were detected more often in *E. coli* from pigs during the metaphylaxis (28/47%, 62/86% and 37/45% strains from P1, P2, P3 respectively) than from sows in the post-exposure period (22/16% from S1 and S2). The nucleotide sequence analysis revealed that all class 1 integron gene cassettes carried TMP resistance genes. The gene cassette array* dfrA1-aadA1* were identified with the highest frequency in *E. coli* from all groups of pigs (26/44%, 59/82%, 35/43%, 12/16%, 9/14%, respectively). The gene cassette* dfrA7* was identified with the lowest frequency (3/4% in *E. coli* from P1, 1/1.2% from P3, 1/1.6% from S2). The *dfrA12-aadA2* gene cassette was detected in 2/3% of *E. coli* from Piglets 1 and in 1/1.2% from Piglets 3. Atypical integrons containing only *int1* and* qacΔE-sul1* genes were detected in six (10%) of *E. coli* from Piglets 1, in two strains (3%) from Piglets 2, and in two (2%) from Piglets 3. The presence of the *int1* gene without other class 1 integron components was revealed in 14 strains (19%) from Piglets 2, 18 (22%) from Piglets 3, and in 23 (30%) and 34 (55%) strains from Sows 1 and Sows 2, respectively.

Class 2 integrons were detected more frequently in strains from Piglets 1 (23/39%) than in isolates from Piglets 2 and Piglets 3 (4/5.5% and 5/6% respectively). Class 2 integrons were detected in 16/21% of the strains derived from Sows 1 and 19/31% of strains from Sows 2. Two cassette arrays, *dfrA1-sat2-aadA1* and *estX-sat2-aadA1*, were detected in class 2 integrons. The gene cassettes *dfrA1-sat2-aadA1* dominated in the isolates from Piglets 1 (36%) and occurred in 2 to 10% of the isolates from other groups of pigs. The *estX-sat2-aadA1* cassette array dominated in *E. coli* from sows and was present in 14/18% and 13/21% *E. coli* from S1 and S2, respectively. *E. coli* from Piglets 1 and 2 carrying this array accounted for only 3 and 4%, respectively. The simultaneous presence of class 1 and class 2 integrons, containing the *dfrA1* gene cassette, was found in 10 (17%) isolates from Piglets 1, and in one strain (1.4%) from Piglets 3.

### 3.4. Resistance Genes Characteristics

Considering the prevalence of the *dfrA1* gene both in integrons and not associated with integrons, the integron location of the *dfrA1* gene predominated in *E. coli* from piglets during the antimicrobial treatment. The *dfrA1* integron gene cassette was detected in 42/76%, 47/66% and 60/73% of strains from Piglets 1, Piglets 2 and Piglets 3, respectively. In isolates from Sows 1 the *dfrA1* integron and non-integron location was similar (15/19%* vs.* 14/18%). In isolates from Sows 2 the *dfrA1* gene not associated with the integrons was more frequently found (15/24% integron and 25/40% non-integron location). The *dfrA7* and *dfrA12* genes occurred as an integron gene cassette only in seven strains from piglets and one from sows.

The coexistence of 2 to 4 TMP resistance genes (associated or not associated with integrons) in various combinations was found in 73 (21%) of all tested isolates. The most frequently appearing genes coexistence concerned the most commonly identified genes: *dfrA1* and *dfrA12* (Table 3). The *dfrA1* gene coexisted also with *dfrA5* or *dfrA7* and *dfrA12* genes. In Piglets 3, combinations of all identified *dfrA* genes were observed (Table 3). Strains carrying more than one SMX resistance gene were detected exclusively in *E. coli* from piglets during medical treatment. Three strains from Piglets 1 and eleven strains from Piglets 3 carried both *sul1* and* sul2* genes. Six strains from Piglets 3 had *sul1* and* sul3* genes.

### 3.5. Plasmid Characteristics

The plasmid characterization was performed for the strains that included multiple TMP resistance genes (n = 71). The plasmid profile for a single strain contained from 1 to 5 plasmids. The plasmid profiles with 2 to 4 plasmids dominated in *E. coli* from animals during the antimicrobial treatment (61%). Overall, plasmids with sizes ranging from 1.5 kb to 100 kb were identified. Large plasmids, of approx. 100, 60 and 12 kb, occurred only in *E. coli* from animals during treatment. The most frequently identified plasmid had size approx. 56 kb, followed by 7 kb and 1.5 kb, and they were isolated from both groups. Plasmids of small size, approx. 8 kb, 6 kb, 4.5 kb and 3.8 kb, were detected only in isolates from pigs after treatment. The replicon typing revealed the presence of eight different plasmid incompatibility groups: FIB, FIIA, K and I1, P, HI1, B/O, T in the set of 71 tested isolates. Replicons FIB, FIIA, K and I1 were detected both in isolates from pigs during the metaphylaxis and in the post-exposure period. Replicons P, HI1, B/O and T were detected only in isolates from pigs during the treatment.

### 3.6. Transcription Analysis of dfrA Genes

In order to determine if all coexisting in a single strains *dfrA* genes contribute to the phenotypic resistance, the transcription analysis of *dfrA* genes was performed. Generally transcription of at least one of the *dfrA* genes was revealed in 31 from 71 tested isolates (Table 4). The presence of the transcript of the *dfrA12* gene was found in 25 out of the 46 strains carrying the gene. In four out of the 22 strains, transcription of the *dfrA7* gene was detected. Also transcription of the *dfrA5* gene was detected rarely, and was revealed in three out of the 18 strains carrying this gene. Transcription of the *dfrA1* gene was not detected in any of the strains carrying this gene although in 28 strains *dfrA1* constituted class 1 integron gene cassette. Only in two cases were the transcripts of two trimethoprim resistance genes in a single strain detected: *dfrA5*,* dfrA7* and *dfrA7*, *dfrA12*. The *dfrA* gene transcription was detected in strains derived both from animals during the antimicrobial therapy and in the post-exposure period (in 16 and 15 strains, respectively).

In order to verify whether the transcription of tested genes can be induced by the direct presence of trimethoprim, Real-time PCR analysis was conducted afresh but with the presence of the antimicrobial in the culture prior to the isolation of RNA. Regardless of the presence of TMP pressure during bacterial growth, the transcription score for each gene in the strain was the same.

**Table 4:** Results of Real-time PCR transcription analyses of *dfrA* genes coexisting in single *E. coli* derived from groups of pigs during metaphylaxis (A+) and in post-exposure period (A−).

Coexisting TMP Resistance Genes	Gene Transcript	No. of *E. coli* from Pigs		Total
		A+	A−	
		(*n* = 46)	(*n* = 25)	(*n* = 71)
*dfrA1, dfrA5*	*dfrA5*	2	0	2
	-	6	1	7
*dfrA1, dfrA7*	-	8	2	10
*dfrA1, dfrA12*	*dfrA12*	11	9	20
	-	11	6	17
*dfrA5, dfrA7*	*dfrA5, dfrA7*	1	0	1
	-	2	0	2
*dfrA5, dfrA12*	*dfrA12*	1	2	3
*dfrA7, dfrA12*	*dfrA7*	0	1	1
*dfrA1, dfrA5, dfrA7*	-	2	0	2
*dfrA1, dfrA7, dfrA12*	*dfrA7*	0	1	1
	*dfrA12*	0	1	1
	*dfrA7, dfrA12*	0	1	1
	-	1	1	2
*dfrA1 , dfrA5, dfrA7, dfrA12*	*dfrA12*	1	0	1
Total		46	25	71

### 3.7. Characteristics of Class 1 Integron Promoters

Gene *dfrA1*, for which no transcript was detected, constituted a part of class 1 integron gene cassettes. Therefore, for all 28 strains carrying class 1 integron with gene cassettes *dfrA1*, the strength revealed in 20 of the tested integrons (compared to gb:KF921585.1). The hybrid weak PcH1 promoter (-35 TGGACA/-10 TAAACT) was detected in eight tested integrons (compared to gb: KF921569.1) detected in *E. coli* from piglets during antimicrobial administration. The simultaneous presence of the second P2 promoter (-35 TTGTTA/-10 TACAGT) was detected in all tested sequences, but without the GGG base sequence in the spacer region [19].

### 3.8. Discussion

The presented research constitutes an assessment of the impact of trimethoprim (TMP) and sulfamethoxazole (SMX) administration in the metaphylactic program on the prevalence and diversity of TMP–SMX resistance in commensal *E. coli* from pigs during the production cycle. Antimicrobial usage in food animals contributes to the development and the spread of resistant microorganisms in the environment. Although antimicrobial growth promoters have been forbidden in the EU since 2006 [20], antimicrobials can be used, apart from the direct treatment of diseases, in control treatment called metaphylaxis. After the diagnosis of infection in a part of a group of animals, antimicrobials are administered to the whole group of animals or to a herd, in order to prevent the spread of disease to animals in close contact and at substantial risk of infection [21,22].

In our study in piglets during the first week of antimicrobial administration (P1) the high prevalence of *E. coli* TMP and SMX resistance was detected. Also all of the tested strains from piglets (P2) taking antimicrobials from 2 and 3 weeks (P3) were resistant. The level of resistance, expressed as MIC values, for all resistant isolates was very high. The high resistance in piglets in the first week of treatment may be due to the rapid development of resistance but also due to the selection of resistant clones. Strains present both in the environment and from the sow can colonize the piglets’ intestine [23,24]. Our study did not cover pigs in the pre-exposure period, but it is highly probable that since the commensal flora of sows is already resistant to antimicrobials, piglets after the birth were colonized by the resistant clones. Horizontal transfer of resistance genes through numerous plasmids detected in the tested strains, may also be responsible for the development of the resistance.

Lower prevalence of resistant *E. coli* was observed in sows that were 10 and 18 weeks after treatment, but the percentage of resistance was not less than 69%. The persistence of resistance in spite of absence of the antimicrobial pressure in the population contributes to the high prevalence of resistance. Different mechanisms can be responsible for the stability of antimicrobial resistance in bacterial populations [25]. In the tested set of isolates from pigs in the post-exposure period, all TMP and SMX resistance genes and integrons that were detected in isolates from pigs during antimicrobial treatment were also observed. It may be the result of adaptation, circulation and maintenance of some set of resistant strains regardless of antimicrobial pressure, in the environment of the studied farm. The differences between the two groups (A+, A−) concerned mainly the number of the detected resistance genes, and more frequent detection of the *estX-sat2-aadA1* gene cassette array in class 2 integrons in *E. coli* from pigs in the post-exposure period. The plasmids of strains from pigs during and after the treatment differed in their sizes and classification to incompatibility groups. Greater diversity was observed in *E. coli* from animals at the time of the metaphylaxis. This indicates the high dynamics of the resistance transfer in the environment during antimicrobial pressure.

In the studies of the genetic background of resistance, the presence of four different TMP resistance genes and three SMX resistance genes was revealed. The *dfrA1*, *dfrA12*, *dfrA7* and *dfrA*5 TMP resistance genes were detected. The general prevalence of the different *dfrA* genes in *E. coli* was in agreement with the earlier studies from different clinical and commensal *E. coli* [26,27]. An exception was the absence of the *dfrA17* gene, because it has been observed frequently by others in *E. coli* isolates from animal origin [26,27,28]. Higher prevalence of the *dfrA1* gene was related to the significant incidence of class 1 and class 2 integrons carrying the *dfrA1* gene cassette detected in this group. The *sul1* and *sul3* genes were the most frequently detected SMX resistance genes among *E. coli* from all groups of pigs, but differences in the proportion of these genes were observed depending on the source of the strains. The *sul1* gene was the most common in *E. coli* from piglets during the antimicrobial therapy, and the *sul3* gene among isolates from animals after the antimicrobial treatment. More frequent occurrence of the *sul1* gene than *sul3* in the *E. coli* from piglets during metaphylaxis was connected with the prevalence of the *sul1* gene in the 3’ conservative segment of class 1 integrons. Class 1 integrons dominated among *E. coli* from this group of pigs. The *sul2* gene was detected with lower incidence in all but the Piglets 3 group. According to many reports, *sul1* gene (as part of an integron) and *sul2* dominate among sulfonamide resistant *E. coli* in humans and animals [17,27,29,30] but also some data show the prevalence of the similar gene *sul3* [16].

The coexistence of multiple TMP and SMX resistance genes in single strains is described in the literature usually with very low frequency [26,28]. In our studies, the coexistence of *dfrA* genes occurred frequently (71/20%) both in *E. coli* from pigs during the antimicrobial treatment and in the post-exposure period. Multiple SMX resistance genes were detected with lower frequency, and concerned 20/9% *E. coli*, exclusively from piglets during treatment. Transcription analysis of the *dfrA* genes revealed that in most cases, only one of the multiple genes underwent transcription. Only in two isolates was the simultaneous transcription of two genes found. The *dfrA12* gene was the most frequently expressed, whereas the *dfrA5* and *dfrA7* genes were expressed in few of the tested isolates. Transcription of these genes was detected in the isolates both from piglets during the treatment and from sows in the post-exposure period. Unexpectedly, the most commonly prevalent *dfrA1* gene was not transcribed in any of the tested isolates. Some of the genes that were not transcribed were associated with integrons. The strength of the promoter is responsible for the level of integron gene cassette transcription [31]; therefore promoter analysis of class 1 integrons was performed. Two different Pc variants were found in combination with the P2 promoter: PcW-P2 and PcH1-P2. Both PcW and PcH1 promoters are classified as weak promoters. It has been demonstrated that when weak Pc and the second P2 promoters are present, the P2 promoter is responsible for 90% of the total promoter activity [32,33,34]. However, the P2 promoter is active when carrying a fragment of 3 bp insertion GGG in its 14-bp spacer. The lack of this GGG base sequence of the P2 promoter in all integrons analyzed in our study was detected. This may be the reason for the absence of the *dfrA* gene cassette transcript in the set of the tested *E. coli*. The presence of the weak promoter PcW, but without a second promoter P2, was previously observed by other researchers of the *dfrA1-aadA1* gene cassette array of class 1 integrons [35]. The reason for the lack of transcription of TMP resistance genes not associated with integrons is not known and requires further detailed research.

It is widely accepted that integrons are a very efficient mechanism for the dissemination of antimicrobial resistance [36,37,38,39,40]. Also the *dfrA1* gene has commonly been detected by many researchers in animal and human *E. coli* isolates [26,30,39], often as an integron gene cassette. However, the reports of unexpressed bacterial resistance genes are rare. Enne and co-workers [41] showed that the expression of plasmid-borne resistance genes could be silenced in the pig gut, while the intact resistance genes and their promoters are retained.

## 4. Conclusions

Great diversity of the genetic background of resistance to TMP and SMX was observed in *E. coli* from animals at the time of metaphylaxis. High resistance levels in the first week of antimicrobials administration confirmed the effect of antimicrobial pressure on the rapid development of resistance and selection of resistant isolates. Also, the persistence of resistance in the absence of antimicrobial pressure in sows, 10 and 18 weeks after treatment, confirmed the statement that once acquired antimicrobial resistance is difficult to lose. In our study, multiple combinations of *dfrA* genes occurred in *E. coli* from all the tested groups. However, a significant portion of these genes was not transcribed, including the most frequently identified *dfrA1* gene in class 1 and 2 integrons. These findings raised the question whether commonly detected genes are responsible for phenotypic resistance.
